# Severe fever with thrombocytopenia syndrome virus trends and hotspots in clinical research: A bibliometric analysis of global research

**DOI:** 10.3389/fpubh.2023.1120462

**Published:** 2023-02-02

**Authors:** Zhengyu Zhang, Juntao Tan, Wen Jin, Hong Qian, Loulei Wang, Hu Zhou, Yuan Yuan, Xiaoxin Wu

**Affiliations:** ^1^Medical Records Department, The First Affiliated Hospital, Zhejiang University School of Medicine, Hangzhou, China; ^2^Operation Management Office, Affiliated Banan Hospital of Chongqing Medical University, Chongqing, China; ^3^Medical Records Department, The First Hospital of Lanzhou University, Lanzhou, China; ^4^General Committee Office, The People's Hospital of Yubei District of Chongqing City, Chongqing, China; ^5^Medical Department, Women and Children's Hospital of Chongqing Medical University, Chongqing, China; ^6^State Key Laboratory for Diagnosis and Treatment of Infectious Diseases, National Clinical Research Centre for Infectious Diseases, The First Affiliated Hospital, Zhejiang University School of Medicine, Hangzhou, Zhejiang, China

**Keywords:** SFTSV, bibliometric, data visualization, CiteSpace, VOSviewer

## Abstract

**Background:**

Since severe fever with thrombocytopenia syndrome virus (SFTSV) was first reported in 2009, a large number of relevant studies have been published. However, no bibliometrics analysis has been conducted on the literature focusing on SFTSV. This study aims to evaluate the research hotspots and future development trends of SFTSV research through bibliometric analysis, and to provide a new perspective and reference for future SFTSV research and the prevention of SFTSV.

**Methods:**

We retrieved global publications on SFTSV from the Web of Science Core Collection (WoSCC) and Scopus databases from inception of the database until 2022 using VOSviewer software and CiteSpace was used for bibliometric analysis.

**Results:**

The number of SFTSV-related publications has increased rapidly since 2011, peaking in 2021. A total of 45 countries/regions have published relevant publications, with China topping the list with 359. The Viruses-Basel has published the most papers on SFTSV. In addition, Yu et al. have made the greatest contribution to SFTSV research, with their published paper being the most frequently cited. The most popular SFTSV study topics included: (1) pathogenesis and symptoms, (2) characteristics of the virus and infected patients, and (3) transmission mechanism and risk factors for SFTSV.

**Conclusions:**

In this study, we provide a detailed description of the research developments in SFTSV since its discovery and summarize the SFTSV research trends. SFTSV research is in a phase of explosive development, and a large number of publications have been published in the past decade. There is a lack of collaboration between countries and institutions, and international collaboration and exchanges should be strengthened in the future. The current research hotpots of SFTSV is antiviral therapy, immunotherapy, virus transmission mechanism and immune response.

## 1. Introduction

Severe fever with thrombocytopenia syndrome (SFTS) virus (SFTSV) is a type of Bunyavirus that is seemingly transmitted by ticks, such as *Haemaphystick longicornis* (1, 2). A significant number of SFTSV are human pathogens that can cause severe diseases such as hepatitis, encephalitis, and hemorrhagic fever (3). SFTSV was firstly detected in 2009 in rural parts of central and northeastern China (4). It has an incubation period of 5–14 days and patients present with clinical symptoms such as fever (>38.6°C), thrombocytopenia, leukopenia, and gastrointestinal symptoms (5). Some severe cases may also present with disturbance of consciousness, skin petechiae, and gastrointestinal and pulmonary hemorrhage (6). In severe cases, secondary encephalopathy and multiple organ failure, fulminant myocarditis, rhabdomyolysis, and hemophagocytic syndrome may develop. This emerging infectious zoonotic disease has also been reported in other Asian countries such as South Korea and Japan (7). In addition, a report in 2012 showed that the clinical manifestations of two cases of new venous virus infection in Missouri, USA were similar to that of SFTSV, and phylogenetic analysis also showed that the virus found in the USA was closely related to SFTSV (8).

The fatality rate among the first infected patients in China was reported to be 30% (7). In response to this emerging infectious disease of unknown cause, in 2010, enhanced surveillance for severe fever with SFTS was implemented. In 2015, the fatality rate for SFTS in Japan and South Korea was over 30% (9). SFTSV can evolve rapidly through genetic mutations and has already become a major threat to public health (3). There is no specific treatment for SFTSV, and the only method to prevent SFTSV infection and transmission is to avoid tick bites (10). Moreover, there is currently no vaccine for SFTSV. Therefore, we must understand the related risk factors and outcomes to strengthen our preparedness strategies.

We performed bibliometric analysis of SFTSV publications published in the Web of Science Core Collection (WoSCC) and Scopus databases. We assessed the number and year of publications, the number of national or regional contributions, the number of journals and disciplines, the number of institutions, the number of international collaborations, and the number of authors, and performed a reference analysis. By analyzing the co-occurrence of SFTSV keywords on a visualization diagram, the SFTSV-related research trends and hotspots were determined, and we hope to encourage advancements in the field through this work. In this report, we review and describe the development and main trends in the published SFTSV literature with a view to providing a new perspective and reference for the future of SFTSV research and SFTSV prevention.

## 2. Materials and methods

### 2.1. Data sources and search strategy

The search terms used were “severe fever with thrombocytopenia syndrome bunyavirus” or “severe fever with thrombocytopenia syndrome virus,” or “SFTS virus,” or “SFTSV,” and the article titles, abstract, and keywords of publications retrieved from the WoSCC and Scopus databases were searched (11, 12). The search period was form January 1, 2010 to November 21, 2022. The document type was limited to “article” and “review article,” and the language was “English.” The three researchers simultaneously examined the articles concerning STFSV by title, abstract, and keywords, and read the full paper if necessary. A total of 648 and 707 publications were identified from the WoSCC and Scopus databases, respectively (2011–2022), see Supplementary Figure 1 for details. The data used in this study came from a public database, so the approval of the ethical committee was not required.

### 2.2. Data analysis and visualization

To ensure the consistency of the results and the reproducibility of this study, two researchers analyzed the data separately. Microsoft Excel 2020 was used to analyze and represent the most productive and cited authors, institutions, journals, and countries/regions. All the data from the WoSCC and Scopus databases were processed using the following functionality of the CiteSpace software: convert to Web of Science plain format and remove duplicates through the dol.list file of the citing article. CiteSpace was used to analyze and display the development context and research hotspots in the STFSV field, and to predict its evolutionary paths, as well as research frontiers (13, 14). The citation bursts for publication year, author, institution, journal, country/region, and keywords were identified and visualized using CiteSpace. The knowledge graph of the parameter “years per slice” was adjusted to 1 year. VOSviewer was used to draw visual knowledge maps for distributions of authors, institutions, and countries/regions, as well as disciplines (15).

In the knowledge graph, different nodes represented various elements such as authors, institutions, countries/regions, and keywords. The size of the nodes reflected the number or frequency of publications, and the larger the node, the higher the number or frequency of publications. The connection lines between the nodes reflected the relationship between the co-operation or co-citation, the thicker the line, the more times of cooperation or co-citation. The different colors of the lines within the nodes represented different times, and the color of the line indicated the years when the co-operation or co-citation first appeared (16–18).

## 3. Results

### 3.1. Trends of publications and citations

**Figure 1:**
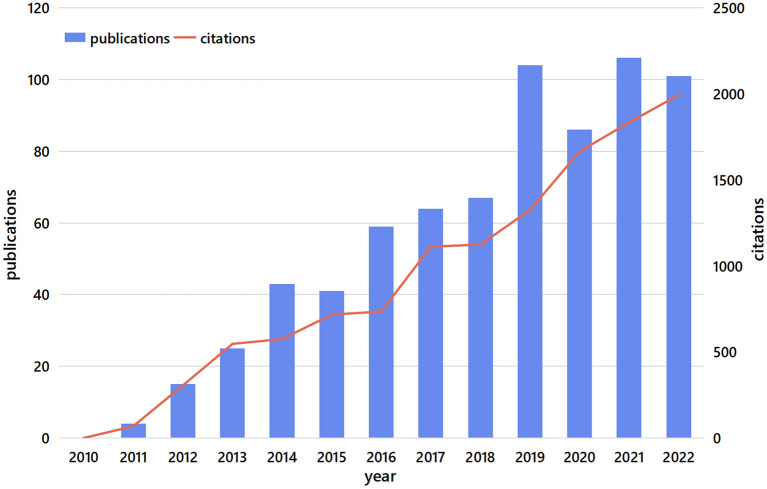
Trend of publications and citations (2011–2022).

The search results were identified by source and duplicates were removed. A total of 715 unique records were obtained, including 672 articles (94.0%) and 43 reviews (6.0%), from WoSCC (594) and Scopus (121). Since 2011, the number of SFTSV-related publications increased rapidly and attained a maximum peak in 2021. The number of citations showed a similar but more stable upward trend compared to the number of publications. The publication in 2022 is less than that in 2021, because this study started in November 2022 and has not finished in 2022, so it is slightly less than that in 2021 (Figure 1).

### 3.2. Contributions of countries/regions

A total of 45 countries/regions have published relevant publications. The Peoples Republic of China ranked first with 359 documents (50.2%), followed by Japan (162, 22.7%) and South Korea (143, 20.0%). The highest total citations of publications were published from The Peoples Republic of China, but the average citations per article was lower than the United States and United Kingdom (Table 1). Total link strength (TLS) indicated the number of connections between nodes. Stronger TLS means more cooperation with other countries. As is shown in Table 1, Peoples Republic of China and the United States were the two countries that carry out the most international cooperation.

**Table 1:** The top 10 countries in number of publications concerning SFTSV.

**Rank**	**Country**	**Total publications**	**Total citations**	**Average citations**	**TLS**
1st	Peoples R China	359	8,085	22.5	105
2nd	Japan	162	2,216	13.7	41
3rd	South Korea	143	1,852	13.0	35
4th	the United States	104	3,129	30.1	101
5th	Germany	17	346	20.4	14
6th	United Kingdom	12	392	32.7	15
7th	France	9	130	14.4	11
8th	India	9	68	7.6	7
9th	Zambia	5	101	20.2	8
10th	Australia	4	11	2.8	6

**Figure 2:**
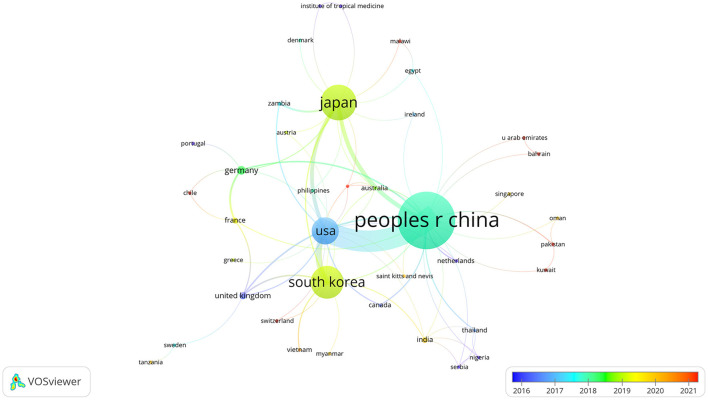
Cooperation map of countries.

When the minimum number of documents for VOSviewer was set to 1, 39 countries/regions met the thresholds. Superimposition of time on the visualized cooperation map of countries/regions is shown in Figure 2. The more purple the color, the earlier the country appeared, and the redder the color, the later the country appeared. The thicker the line is, the stronger the cooperation. The top four total link strength countries/regions were The Peoples Republic of China, the United States, Japan, and South Korea. The Peoples Republic of China had cooperated with numerous countries/regions in SFTSV-related research, and their most significant cooperations were with the United States and Japan.

### 3.3. Contributions of top journals

All the publications were published in a total of 207 journals, and 34 journal published at least five articles, with the top 10 journals accounting for 33.3% of the total publications included in this analysis. Among the top 10 publication journals on SFTSV, Viruses-Basel (IF = 5.818) ranked highest with ~34 articles, followed by Emerging Infectious Diseases (IF = 16.126, 31), and Journal of Virology (IF = 6.549, 29). Journal of Virology (IF = 6.549), Emerging Infectious Diseases (IF = 16.126, 31), and Journal of Infectious Diseases (IF = 7.759) were the top 3 ranked cited journals with 1,586, 1,352, and 807 co-citations, respectively (Table 2).

**Table 2:** The top 10 journals in number of publications and citations concerning SFTSV.

**Rank**	**Publication journal**	**Documents**	IF^*^	**Citations**	**Cited journal**	**Documents**	IF^*^	**Co-Citations**
1st	Viruses-Basel	34	5.818	297	Journal of Virology	29	6.549	1,586
2nd	Emerging Infectious Diseases	31	16.126	1,332	Emerging Infectious Diseases	31	16.126	1,352
3rd	Journal of Virology	29	6.549	1,199	Journal of Infectious Diseases	6	7.759	807
4th	PLoS Neglected Tropical Diseases	28	4.781	369	New England Journal of Medicine	1	176.077	772
5th	PLoS ONE	24	3.752	498	Clinical Infectious Diseases	5	20.999	685
6th	Ticks and Tick-Borne Diseases	23	3.817	377	PLoS ONE	24	3.752	579
7th	American Journal of Tropical Medicine and Hygiene	19	3.707	375	Proceedings of the National Academy of Sciences of the United States of America	4	12.778	411
8th	Scientific Reports	17	4.996	233	American Journal of Tropical Medicine and Hygiene	19	3.707	411
9th	Frontiers in Microbiology	17	6.064	111	PLoS Neglected Tropical Diseases	28	4.781	388
10th	Japanese Journal of Infectious Diseases	16	2.541	188	PLoS Pathogens	9	7.464	329

^*^Impact factors (IF) based on Clarivate Analytics ‘Journal Citation Reports (JCR) 2021.

**Figure 3:**
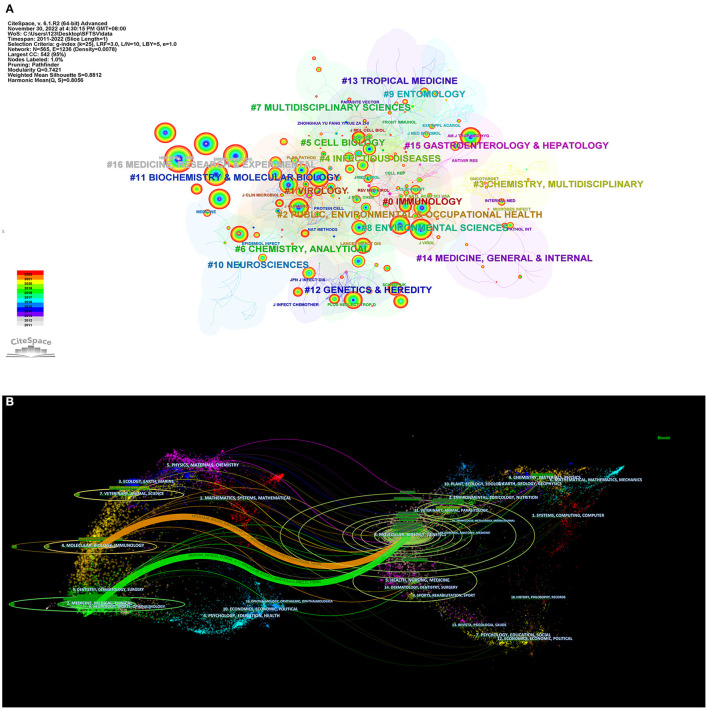
Contributions of top journals on SFTSV. **(A)** Journal co-citation network of clustering subjects categories. **(B)** A dual-map overlap of journals on SFTSV research by citespace (distribution of cited literature and journals on the left and articles that cited literature on the right).

The journals that published SFTSV-related articles were mainly from the immunology, virology, public, environmental and occupational health fields. The different colors of the circles indicate the number of citations of the journals in different years, with purple to red representing the year of the citation from oldest to most recent (Figure 3A).

The dual map of the journals shows that there were two paths for citing and cited journals: (1) Molecular, Biology and Immunology-Molecular, Biology, Genetics; and (2) Medicine, Medical, Clinical-Molecular, Biology, Genetics. The citing journals were mainly concentrated in four circles including six fields: (1) Chemistry, Materials, Physics; (2) Veterinary, Animal, Parasitology; (3) Molecular, Biology, Genetics; (4) Health, Nursing, Medicine; (5) Dermatology, Dentistry, Surgery; and (6) Sports, Rehabilitation, Sport (Figure 3B).

### 3.4. Analysis of institution and co-institution

**Figure 4:**
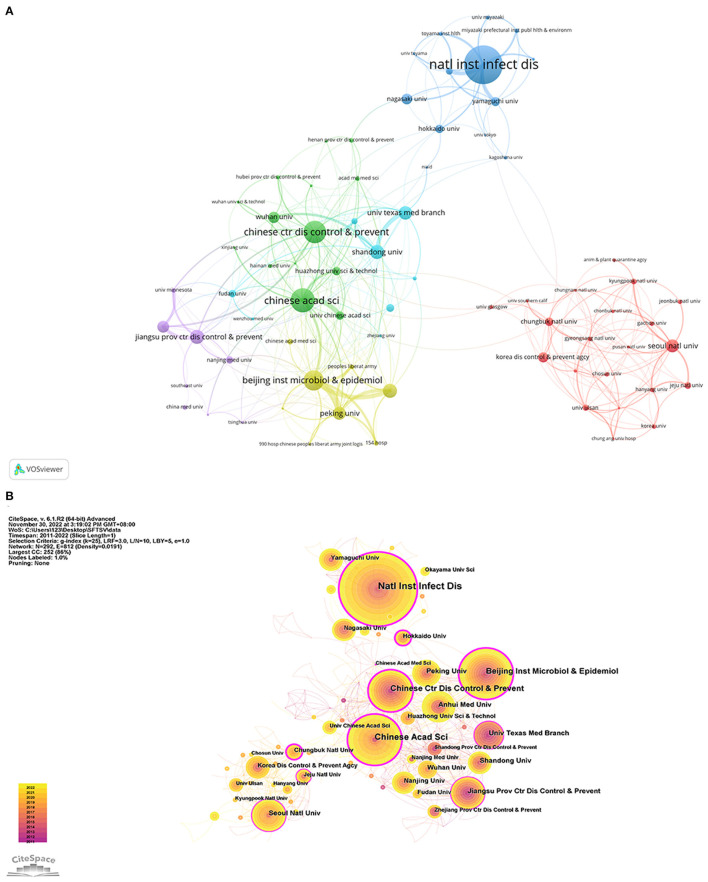
Visualization map of institution on SFTSV. **(A)** Cooperation map of 72 institutions with the number of publications no less than 5 times. **(B)** Centrality cooperation map of institutions.

The “institution” node analysis showed that the institution with the largest number of SFTSV-related articles published was the National Institute of Infectious Disease (Japan), and seven of the top 10 research institutions were located in The Peoples Republic of China (Table 3). The VOSviewer parameters were set to the minimum number of documents for an institution = 5, and 72 institutions were obtained. As shown in Figure 4A, different colors represent different collaboration clusters, almost all of which were conducted within individual countries, with South Korea in red, Japan in blue, and Peoples Republic of China in green, yellow and purple.

**Table 3:** The top 10 institution in number of publications concerning SFTSV.

**Rank**	**Institution**	**Countries/regions**	**Documents**	**Citations**	**Centrality**
1st	National Institute of Infectious Diseases	Japan	97	1,251	0.25
2nd	Chinese Academy of Sciences	Peoples R China	58	1,434	0.35
3rd	Chinese Center for Disease Control and Prevention	Peoples R China	53	2,108	0.41
4th	Beijing Institute of Microbiology and Epidemiology	Peoples R China	47	1,033	0.21
5th	Shandong University	Peoples R China	32	1,032	0.07
6th	Jiangsu Provincial Center for Disease Control and Prevention	Peoples R China	32	1,037	0.13
7th	Anhui Medical University	Peoples R China	32	525	0.04
8th	University of Texas Medical Branch	the United States	30	1,579	0.15
9th	Peking University	Peoples R China	29	426	0.05
10th	Seoul National University	South Korea	29	656	0.15

CiteSpace was used to construct the co-institutions knowledge map (Figure 4B). The purple outermost circle represents the betweenness centrality (BC) (19), which indicates the significance of the nodes in networks. The larger the purple circle, the greater the BC, which is an indicative measure of the contribution of research achievements of institutions to the SFTSV field. Chinese Academy of Sciences (BC = 0.41), Chinese Center for Disease Control and Prevention (BC = 0.35), and Chungbuk National University (BC = 0.32) occupied important positions in the cooperation network.

### 3.5. Analysis of author

**Figure 5:**
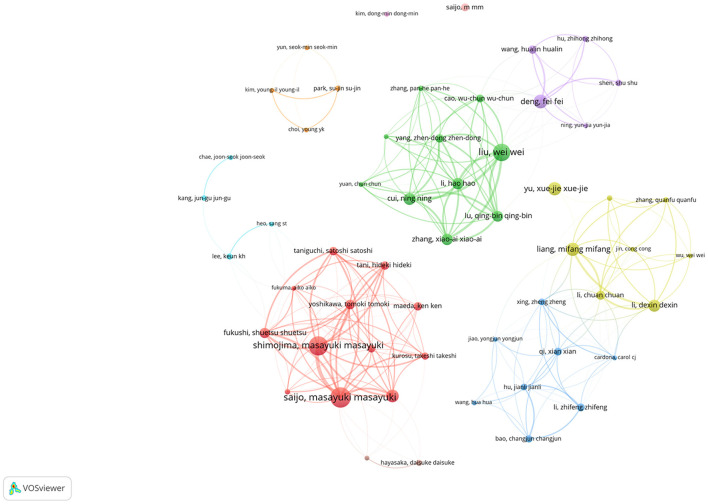
Cooperation map of 55 authors with the number of publications no less than 5 times.

The top three SFTSV-related researchers with the largest number of published articles were Saijo, Shimojima, and Liu Wei, with 43, 40, and 36 articles respectively (Table 4). SFTSV-related researchers with more than 10 published articles were included in the network of co-authorship. Cooperations among authors were divided into clusters with different colors based on co-authorship analysis. As shown in Figure 5, the 55 authors were divided into 10 clusters with different colors. The connection lines between the different clusters were very thin or had no connection lines, indicating little or no cooperation between the clusters.

**Table 4:** The top 10 authors in number of publications concerning SFTSV.

**Rank**	**Author**	**Countries/ regions**	**Documents**	**Citations**
1st	Saijo Masayuki	Japan	43	829
2nd	Shimojima Masyuki	Japan	40	703
3rd	Liu Wei	Peoples R China	36	861
4th	Deng Fei	Peoples R China	29	555
5th	Liang Mifang	Peoples R China	28	1,453
6th	Yu Xuejie	Peoples R China	28	1,163
7th	Morikawa Sshigeru	Japan	27	519
8th	Cui Ning	Peoples R China	26	80
9th	Li Dexin	Peoples R China	25	1,339
10th	Li Hao	Peoples R China	24	522

### 3.6. Analysis of reference

**Figure 6:**
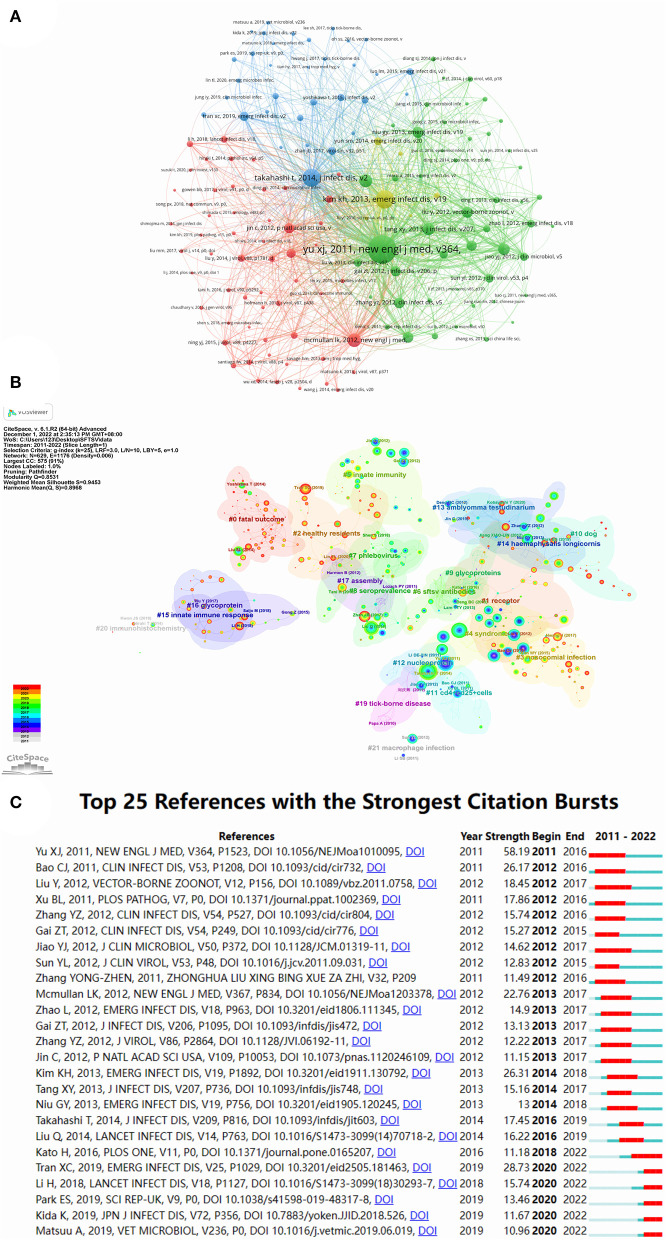
Visualization map of reference on SFTSV. **(A)** Distribution of 148 reference with a frequency of no less than 20 times. **(B)** Reference co-citation network for clustering title terms. **(C)** Top 25 references with the strongest citation bursts.

Using cited reference analysis of VOSviewer, the minimum number of documents of a cited reference was set at 20, and 148 met the threshold. The cited reference network with four clusters is shown in Figure 6A. McMullan et al. (8), Kim (20), Yu et al. (21), and Takahashi et al. (22) had the highest total citations in the red, yellow, green, and blue clusters respectively. Yu et al.'s (21) article had the largest number of co-citations, indicating that this article is the most important research achievement in the SFTSV field (Table 5).

**Table 5:** The top 10 reference in number of co-citations concerning SFTSV.

**Rank**	**Author**	**Journals**	**DOI**	**Year**	**Co-citation**
1st	Yu XJ	New Engl J Med	10.1056/NEJMoa1010095	2011	502
2nd	Tim KH	Emerg Infect Dis	10.3201/eid1911.130792	2013	260
3rd	Takahashi T	J Infect Dis	10.1093/infdis/jit603	2014	250
4th	Mcmullan LK	New Engl J Med	10.1056/NEJMoa1203378	2012	167
5th	Liu Q	Lancet Infect Dis	10.1016/S1473-3099(14)70718-2	2014	159
6th	Tang XY	J Infect Dis	10.1093/infdis/jis748	2013	144
7th	Bao CJ	Clin Infect Dis	10.1093/cid/cir732	2011	135
8th	Liu Y	Vector-Borne Zoonot	10.1089/vbz.2011.0758	2012	131
9th	Niu GY	Emerg Infect Dis	10.3201/eid1905.120245	2013	126
10th	Xu BL	PLoS Pathog	10.1371/journal.ppat.1002369	2011	124

A co-cited references cluster analysis was conducted using CiteSpace with set node type = cited reference, and other parameters were set to their default values. We obtained 22 clusters that clearly demonstrated the research themes in the SFTSV field (Figure 6B), and different colors from gray to red represented the number of co-citations in different years. Figure 6C presents the top 20 references with the strongest citation bursts. “Fever with thrombocytopenia associated with a novel bunyavirus in China,” which began in 2011, was the strongest citation burst with an intensity of 58.19.

### 3.7. Analysis of keywords

**Figure 7:**
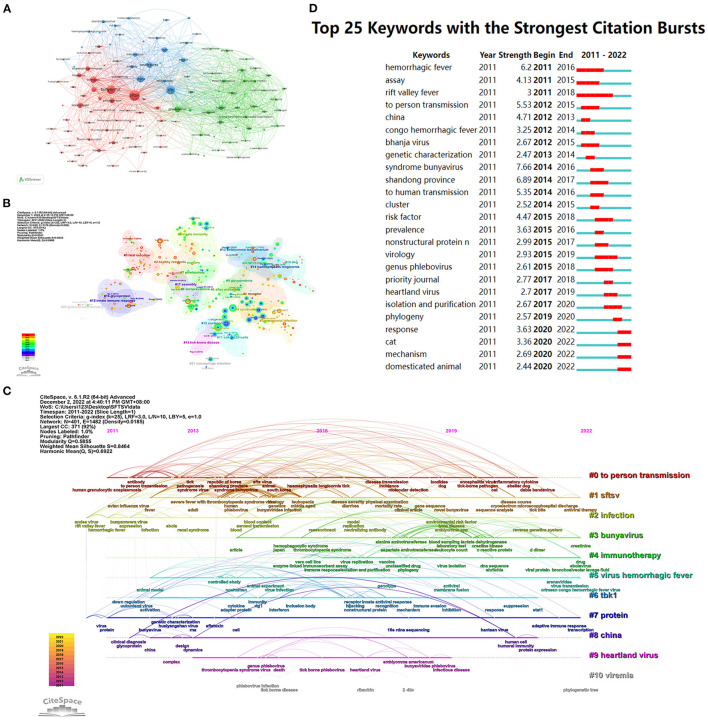
Visualization map of keywords on SFTSV. **(A)** Distribution of 121 keywords with a frequency of no less than 20 times. **(B)** Keywords co-occurrence network. **(C)** Timeline view of keyword cluster. **(D)** Top 25 keywords with the strongest citation bursts.

Keyword co-occurrence analysis was used to determine the main directions and hotspots in SFTSV-related research. In the co-occurrence analysis in VOSviewer, more than 121 keywords occurred more than 20 times. The keyword co-occurrence network map of three different color clusters shows the main directions in SFTSV research. The keywords in the red cluster were mainly pathogeneses and symptoms, the green cluster was related to the characteristics of the virus and infected patients, and the blue cluster was the transmission mechanism and high-risk factors in SFTSV (Figure 7A).

CiteSpace performed cluster calculation according to the co-occurrence of keywords, and obtained 11 clusters, as listed in Supplementary Table 1. The modularity Q = 0.5855 and mean silhouette S = 0.8464, which was considered high, meaning that the network was reasonably divided into loosely coupled clusters (Figure 7B). The keywords of 11 clusters were described along the horizontal timeline in the timeline visualization, showing the research progress in the SFTSV field from 2011 to 2022 (Figure 7C). New keywords appeared in different clusters from 2021 to 2022; for example: Arenaviridae, virus transmission, and Crimean Congo hemorrhagic fever virus appear in #5 virus hemorrhagic fever; creatinine and platelet count in #3 bunyavirus; adaptive immune response and transcription in #7 protein, which may indicate new direction in SFTSV research. Citation bursts are terms that occur abruptly or increased dramatically in frequency in a short period of time, indicating the evolution of the research hotspot over time. The citation bursts in this discipline began in 2011, and 25 keywords with the strongest citation bursts from 2011 to 2022. The strongest citation bursts were syndrome bunyavirus with 7.66 strength from 2014 to 2016. Four keywords (response, cat, mechanism, and domesticated animal) appeared at the end of 2022, and most probably represent hotspots in current SFTSV research (Figure 7D).

## 4. Discussion

We found a total of 715 unique published articles on SFTSV research across two databases. The number of publications showed a significant upward trend since 2011. This trend may be related to the widespread epidemic of SFTSV in many countries/regions in Asia after 2010 (23). The first case of SFTSV was reported from The Peoples Republic of China in 2009, and further cases have been reported in many countries in Asia over the past 10 years, including South Korea and Japan (2013), Vietnam (2017), Myanmar (2018), Taiwan region (2019), and Thailand and Pakistan (2020) (21, 22, 24–29). Since the first case report, SFTSV has attracted great public health attention in Asia, due to the large number of case reports and its high initial case fatality rate of 12% to 30% (21, 30).

The Peoples Republic of China had the largest number of SFTSV cases reported and was the most widespread. A total of 13,824 SFTSV cases (8,899 laboratory-confirmed cases and 4,925 probable cases) had been reported in The Peoples Republic of China, and the regional distribution of SFTSV in The Peoples Republic of China had gradually expanded from five provinces in 2010 to 25 provinces in 2019 (30). The Peoples Republic of China was the main contributor and the most important cooperative partner in the field of SFTSV globally. The Peoples Republic of China had published the most articles in the SFTSV field in the past decade or more (359 as of November 2022). The Peoples Republic of China had cooperated with many countries in SFTSV research, as shown in Figure 2. The United States is the most important cooperative country for The Peoples Republic of China in SFTSV-related research, mainly related to the Heartland Virus, a virus similar to SFTSV isolated from two independent patients in northwest Mississippi in the United States in 2012 (8). In this study, SFTSV-related articles were mainly published in four countries, namely, The Peoples Republic of China, Japan, South Korea, and the United States. However, although Japan and South Korea ranked the third and fourth with 162 and 143 articles published, respectively, and the total citations and the average citations were much lower than those in the Peoples Republic of China and the United States, indicating that Japan and South Korea lack high-quality articles in SFTSV research.

The research findings of collaborations were also influenced by country. The Peoples Republic of China had seven of the top 10 institutions (Table 3). A number of universities, research laboratories, and even government departments located in the Peoples Republic of China had made contributions to SFTSV research, which demonstrates that Chinese institutions attach great importance to SFTSV. The Chinese Academy of Sciences and the Chinese Center for Disease Control and Prevention, which are located in the Peoples Republic of China play the most important role in the SFTSV field. The collaborative network of institutions and authors is loose, and a large number of studies are conducted mainly within one country, illustrating the lack of international collaboration in SFTSV research. As SFTSV continues to be reported and valued in countries around the world, we believe that the institutions and authors from Asian countries should cooperate more extensively and closely.

Journals with more citations and co-citation frequencies play an important role in the SFTSV field. However, not all high-yield journals have high numbers of citations. The journal with the most published articles was Viruses-Basel, but the total citations was only 297, indicating that articles from this journal were not the main choice for most SFTSV-related researchers. The citations and co-citations of the Journal of Virology and Emerging Infectious Diseases were among the top 3, indicating that these two journals have published high-quality publications with convincing results. Table 2 lists the top 10 high-yield journals and high co-citation journals for SFTSV-related research, and academic publications on SFTSV may be preferentially published in high-yield journals, while high co-citation journals had published mature research results. There are only two paths between the cited journal and the citing journal, which means that advancement in the SFTSV field will require more cross-disciplinary collaboration.

Yu's (21) article, “Fever with thrombocytopenia associated with a novel bunyavirus in China” published in the New England Journal of Medicine in 2011 had the highest number of co-citations. The article introduced the process of isolating the virus from the blood sample, identified the family of the virus based on the RNA sequence analysis, and named it SFTS bunyavirus. The presence of the virus was confirmed from 171 patients by detection of viral RNA or specific antibodies to the virus in the blood, or both. This was the first systematic article on the isolation and diagnosis of SFTSV and is the most important research achievement in the SFTSV field.

Keyword frequency may reflect the development tendency of research hotspots from another point of view. VOSviewer divided 715 previous studies into three categories according to keywords: (1) pathogenesis and symptoms, (2) characteristics of the virus and infected patients, and (3) transmission mechanism and risk factors of SFTSV.

The main clinical features of SFTS on presentation include fever, thrombocytopenia, leukocytopenia, and gastrointestinal symptoms (31). Before 2010, SFTSV was assumed to be caused by bacteria such as *Anaplasma phagocytophilum* bacterium, due to the fact that no pathogens had been isolated from patients (32). Virus isolation is the commonly employed method in research institutions and it provides sufficient evidence of SFTSV infection, but it is very time-consuming. Amplification of viral nucleic acid and the reverse transcription PCR method are frequently used for clinical confirmation (33).

According to the pathogenicity of the bunyavirus, SFTSV prevents the host's immune response and is manifested through intense virus replication along with multiple organ failure (34). Examination of SFTSV patients has revealed that the number of natural killer cells increases, mainly during the acute phase and grievous SFTSV infection (35). Natural killer cells play an immunomodulatory function by producing various cytokines, and the number of these cytokines is proportional to the severity of the disease. Natural killer cells perform their immunomodulatory functions by producing various cytokines, the amount of which is proportional to the severity of the disease. These cytokines play important roles in serum virus load and other related clinical characteristics. Monocyte chemotactic protein 1 and interleukin-8 are crucial in progressive kidney injury (36), monocyte chemotactic protein 1 and interferon-c-inducible protein produce liver inflammation with fibrosis (37), and interleukin-8 can raise capillary permeability (38).

Much of the SFTSV research focused on “to person transmission” SFTSV is an infectious phlebovirus with a high mortality rate. It mainly affects humans, and although SFTSV can be transmitted through infected animals, there have been no reported cases of SFTSV infection in animals. Vertebrates are the host of this disease and ticks function as vectors, where the virus can undergo brisk changes using gene mutation, homologous recombination, and reassortments. Although the mechanism of SFTSV transmission remains unclear, a large number of studies have suggested that ticks may be the transmission vectors and that domestic or wild animals may be amplifying hosts (39). Age was considered to be a critical risk factor for morbidity and mortality in SFTS, which mainly targets people over 50 years of age. Farmers were the main high-risk population, and there were more female cases than male cases (30).

Based on the timeline viewer, we combined the keywords cluster with the relevant article for analysis to find the recent hotpots of SFTSV-related research. Five clusters emerged with new keywords in 2022.

Antiviral therapy in #1 SFTSV. Zhang et al.'s review (40) suggested that broad-spectrum antivirals have the potential to be the first line of defense to prevent the progression of the disease, and fapiravir maybe the most promising treatment. The future development of antiviral methods may depend mainly on targeted therapies such as monoclonal antibodies and prevention through vaccination.

Drug, ebolavirus, and bronchoalveolar lavage fluid in #4 immunotherapy. An approved drug (tilorone) was highly effective in the treatment of SFTSV infection and may have the potential to be a “universal vaccine” for antiviral infections (41). Małkowska et al. (42) reported that RIG-I-like receptors are promising in the treatment of viral hemorrhagic fevers. Bronchoalveolar lavage fluid is one of the therapeutic tools available to combat viral diseases (ebolavirus, SFTSV), and one reported case of aspergillus isolated in bronchoalveolar lavage fluid showed that invasive fungal disease may accompany the early clinical course of SFTSV infection (43, 44).

Crimean Congo hemorrhagic fever (CCHFV), virus transmission, and Arenaviridae in #5 virus hemorrhagic fever. Teng et al. (45) evaluated the environmental suitability and transmission risk of major Bunyavirales viruses in China by mapping the geographical distribution of all 89 Bunyavirales viruses reported in China from January 1951 to June 2021, and highlighted that Hantaviruses, *Dabie bandavirus*, and CCHFV had the severest disease burden. Two viruses, CCHFV and Rift valley fever virus (RVFV), may occur in local area outbreaks in China. Xinjiang and southwestern Yunnan had the highest environmental suitability to CCHFV occurrence, and southern China had the highest environmental suitability to RVFV transmission all year round.

Adaptive immune response and transcription in #7 protein. Wang et al. (46) summarized the mechanism of SFTSV evasion of the host immune response and highlighted that SFTSV can escape from host immune responses *via* multiple strategies, such as interfering with the number and function of innate and adaptive immune cells, inhibiting the inhibiting interferon signaling pathway, regulating the NF-κB signaling, and autophagy. Moreover, a proposed strategy against the virus may be to regulate the host dysfunctional immune cells. Lan et al. (47) identified two clicks from the Lassa virus microgenome (MG) system, F1204 and F1781, that effectively inhibited authentic lymphocytic choriomeningitis virus (LCMV) and SFTSV infections.

Phylogenetic tree in #10 viremia. Xu et al. (32) constructed a phylogenetic tree based on the M segments of SFTSV, which was epidemic in the Jiaodong area of the Shandong Province, and found local endemic strains were mainly C2 and C3 isolates of SFTSV, and epidemic strains showed relatively stable heredity.

In terms of the top keywords with the strongest citation bursts, response, cat, mechanism, and domesticated animal appeared at the end of 2022 (Figure 7D). We believe that the anti-SFTSV immune responses, molecular mechanism, and viral transmission from animals may represent the future direction of SFTSV research.

Our bibliometrics have certain limitations inherents. First, CiteSpace and VOSviewer are used for bibliometric analysis in this paper, and only analyzed the main conclusions rather than full text, which cannot completely replace system search. Secondly, the data we retrieved were all from WoSCC and Scopus, which are considered to be the most commonly used databases for bibliometric analysis. Although the data from WoSCC and Scopus could be representative of numerous information to a certain extent, some documents excluded from these two databases should be taken into consideration, and the search strategy is designed to favor the matching accuracy of the search, and citation counts were probably underestimated. Finally, in this study, we mainly utilized a quantitative analysis approach, and paid little attention to qualitative research. As a result, certain critical points and details may be missed. Nevertheless, in this study, all the maps based on the retrieved data can intuitively present the hotspots, evolution process and development trend of SFTSV, which could providing many important reference values for newcomers in this field.

## 5. Conclusions

Through bibliometric analysis and data visualization, we elucidated on the research progress, research hotspots, and research frontier in the SFTSV field. We have identified the countries, journals, institutions, authors, and representative publications that play an important role in this field. The findings inform investigations on SFTSV and identify the potential partners for interested researchers. Our study found that SFTSV research is in a phase of explosive development, and a large number of publications have been published in the past decade. There is a lack of collaboration between countries and institutions, and international collaboration and exchanges should be strengthened in the future. The research direction of SFTSV is on the therapeutic strategy of SFTSV, the transmission mechanism of the virus, and the immune response. The current research hotpots of SFTSV is antiviral therapy, immunotherapy, virus transmission mechanism and immune response.

## Data availability statement

The raw data supporting the conclusions of this article will be made available by the authors, without undue reservation.

## Author contributions

ZZ and JT designed the research. ZZ, WJ, HQ, and HZ collected and organized data. ZZ, YY, and LW analyzed the data. ZZ, JT, and XW drafted the manuscript. YY and XW contributed to the critical revision of the manuscript. All authors contributed to the manuscript and approved the submitted version.
