# Supplementary material for: Severe fever with thrombocytopenia syndrome virus trends and hotspots in clinical research: A bibliometric analysis of global research
Source: Front Public Health. 2023 Feb 2;11:1120462. doi: 10.3389/fpubh.2023.1120462 (PMC9933999; doi:10.3389/fpubh.2023.1120462)
Supplement: Supplementary file 1 [file Table_1.DOCX]

**Supplementary information**

**Figure S1. Flowchart of the study strategy**


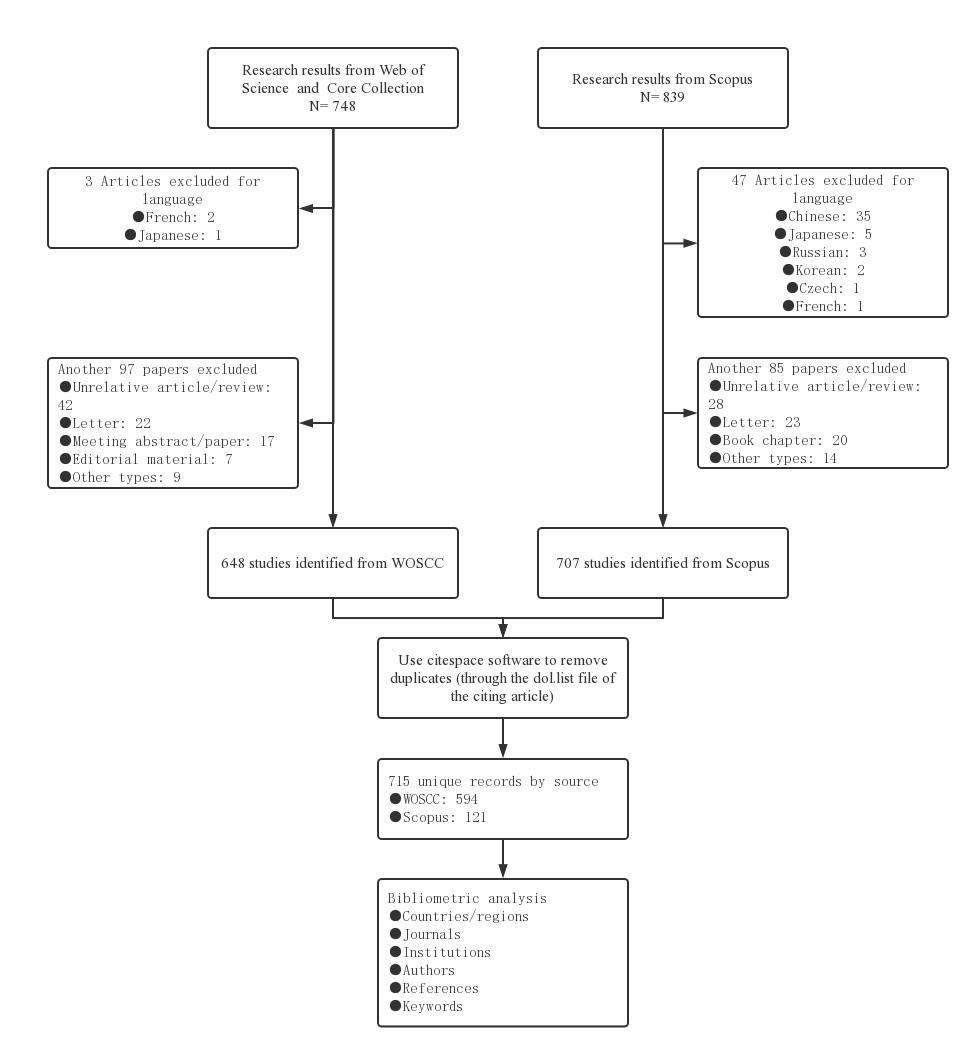


**Table S1. Cluster list of keywords co-occurrence**

| **Cluster** | **Size** | **Silhouette** | **Mean(Year)** | **Main keywords in cluster** |
| --- | --- | --- | --- | --- |
| 0# to person transmission | 68 | 0.753 | 2015 | to person transmission; sfts virus; syndrome virus; south korea |
| 1# sftsv | 63 | 0.886 | 2016 | sftsv; bunyavirus; fever; human; infection |
| 2# infection | 34 | 0.913 | 2014 | infection; molecular diagnosis; expression; human; bunyavirus |
| 3# bunyavirus | 34 | 0.767 | 2019 | bunyavirus; aspartate aminotransferase; alanine aminotransferase |
| 4# immunotherapy | 33 | 0.853 | 2018 | immunotherapy; virus replication; south korea; vaccine |
| 5# virus hemorrhagic fever | 33 | 0.918 | 2015 | virus hemorrhagic fever; bunyavirus; nonhuman; controlled study |
| 6# tbk1 | 31 | 0.801 | 2016 | tbk1; immunity; ikk epsilon; cytokine |
| 7# protein | 24 | 0.862 | 2014 | protein; sftsv; bunyavirus; cell |
| 8# china | 23 | 0.893 | 2016 | china; clinical diagnosis; humanoral immunity |
| 9# heartland virus | 21 | 0.903 | 2015 | heartland virus; thrombocytopenia syndrome virus; tick borne phlebovirus; genus phlebovirus |
| 10# viremia | 7 | 0.916 | 2018 | viremia; tick borne disease; ribavirin |
